# Antimicrobial resistance among GLASS pathogens in Morocco: an epidemiological scoping review

**DOI:** 10.1186/s12879-022-07412-4

**Published:** 2022-05-07

**Authors:** Chakib Nejjari, Youness El Achhab, Amina Benaouda, Chakib Abdelfattah

**Affiliations:** 1 International School of Public Health, Mohammed VI University of Health Sciences (UM6SS), Casablanca, Morocco; 2 Laboratory of Epidemiology, Clinical Research and Community Health, Faculty of Medicine and Pharmacy of Fez, University Sidi Mohamed Ben Abdellah, Km 2.2 Rte Sidi Harazem, B.P 1893, Fez, Morocco; 3 CRMEF Fez-Meknes, Rue Kuwait, B.P 49, Fez, Morocco; 4 Department of Microbiology, Cheikh Zayed International University Hospital, Rabat, Morocco; 5 Department of Infectious Diseases, Faculty of Medicine, University Hassan II, Casablanca, Morocco

**Keywords:** Antimicrobial resistance, Bacteria, Morocco, Global antimicrobial resistance surveillance system, Scoping review

## Abstract

**Background:**

Monitoring of antimicrobial resistance (AMR) is of great importance due to the frequency of strains becoming increasingly resistant to antibiotics. This review, using a public health focused approach, which aims to understand and describe the current status of AMR in Morocco in relation to WHO priority pathogens and treatment guidelines.

**Methods:**

PubMed, ScienceDirect and Google Scholar Databases and grey literature are searched published articles on antimicrobial drug resistance data for GLASS priority pathogens isolated from Morocco between January 2011 and December 2021. Articles are screened using strict inclusion/exclusion criteria. AMR data is extracted with medians and IQR of resistance rates.

**Results:**

Forty-nine articles are included in the final analysis. The most reported bacterium is *Escherichia coli* with median resistance rates of 90.9%, 64.0%, and 56.0%, for amoxicillin, amoxicillin-clavulanic acid, and co-trimoxazole, respectively. Colistin had the lowest median resistance with 0.1%. A median resistance of 63.0% is calculated for amoxicillin-clavulanic acid in *Klebsiella pneumonia*. Imipenem resistance with a median of 74.5% is reported for *Acinetobacter baumannii*. AMR data for *Streptococcus pneumonie* does not exceed 50.0% as a median.

**Conclusions:**

Whilst resistance rates are high for most of GLASS pathogens, there are deficient data to draw vigorous conclusions about the current status AMR in Morocco. The recently join to the GLASS system surveillance will begin to address this data gap.

**Supplementary Information:**

The online version contains supplementary material available at 10.1186/s12879-022-07412-4.

## Background

Antimicrobial resistance (AMR) is increasingly recognized as a global public health issue by leading to a high rate of morbidity and mortality [1, 2]. By 2050, the global mortality will have attributed to AMR that could reach 10 million per year; this will pose a significant threat to the global economy if measures are not taken to curb the problem [3]. The antimicrobials misuse and abuse in veterinary and human medicine have accelerated the growing worldwide phenomenon of AMR [4–6]. Moreover, the use of antimicrobials in the food chain endangers sustainable food production and food security [7].

In October 2015, the World Health Organization (WHO) launched the Global Antimicrobial Resistance Surveillance System (GLASS), as a necessary contribution to the global action plan against AMR. Morocco joined GLASS system by the end of 2018 [8]. Recent AMR data collected from two million patients over 66 countries show high rates of resistance among antimicrobials frequently used to treat common bacterial infections [9]. The main AMR profiles are defined as those identified by WHO as “priority pathogens” for the public health significance. There are eight organisms: *Escherichia coli*, *Klebsiella pneumoniae*, *Acinetobacter baumannii*, *Staphylococcus aureus*, *Streptococcus pneumoniae*, *Salmonella* spp*.*, *Shigella* spp*.*, and *Neisseria gonorrhoeae* [9] (Additional file 2: Appendix 1). Pathogen-antimicrobial combinations under GLASS surveillance include penicillins, third- and fourth-generation cephalosporins, carbapenems, fluoroquinolones, aminoglycosides, tetracyclines, polymyxins, macrolides, and co-trimoxazole.

It is well known that *E. coli* and *K. pneumoniae* are the most common pathogens of urinary tract infections (UTIs), which are one of the most common bacterial infections [10–13]. Uropathogenic *E. coli* strains have a range of adhesins that allow the bacteria to aggregate and adhere to the cellular surfaces [11, 14]. In addition to UTIs, *K. pneumonia* causes a variety of infectious diseases, including bacteremia, pneumonia, and liver abscesses. *K. pneumoniae* multidrug-resistant strains are closely related to the antibiotic resistance genes encoded by plasmid [15, 16]. The extensive use and misuse of carbapenems to treat diseases and infections caused by multidrug-resistant gram-negative bacteria contribute to the evolution of plasmid-mediated carbapenemases [17]. *A. baumannii* and *S. aureus* are some of the more common opportunistic pathogens which cause community and nosocomial infections. Unfortunately, the number of multidrug-resistant *A. baumannii* isolates has increased significantly [18, 19]. Resistance to antibiotics is widespread in *S. aureus*, which methicillin-resistant *S. aureus* (MRSA) are the most important clinically [20].

*S. pneumoniae* is an opportunistic pathogen causes pneumonia, meningitis, sepsis, bacteremia, and otitis media, especially in individuals with underdeveloped, weakened, and or deteriorating immune systems. *S. pneumoniae* has developed increased resistance to multiple classes of antibiotics [21, 22]. *Salmonella* belongs to the family Enterobacteriaceae and causes especially gastroenteritis, bacteraemia and enteric fever [23]. Antimicrobial resistance in *Salmonella* strains is a serious health problem worldwide. Mechanisms of *Salmonella* resistance are related especially to genes encoding proteins related to drug transport [24, 25]. *Shigella* causes especially acute gastrointestinal infections and is increasingly becoming highly drug resistant [26–28]. In the same line, *Neisseria gonorrhoeae* has developed resistance to every antibiotic currently approved for treatment [29].

In Morocco, recently, the Ministry of Health creates the national coordination unit and the technical committee for the surveillance of AMR. However, earliest studies highlighted the resistance seriousness of microorganisms to antibiotics [30–32]. Hitherto, however, these data have not been combined to provide a perspective at a national level. This review aims to describe the recent published AMR data from Morocco and gives a summary of key AMR patterns in the country by focusing on the organisms identified by WHO-GLASS.

## Methods

### Sources of information and search strategies

**Fig. 1:**
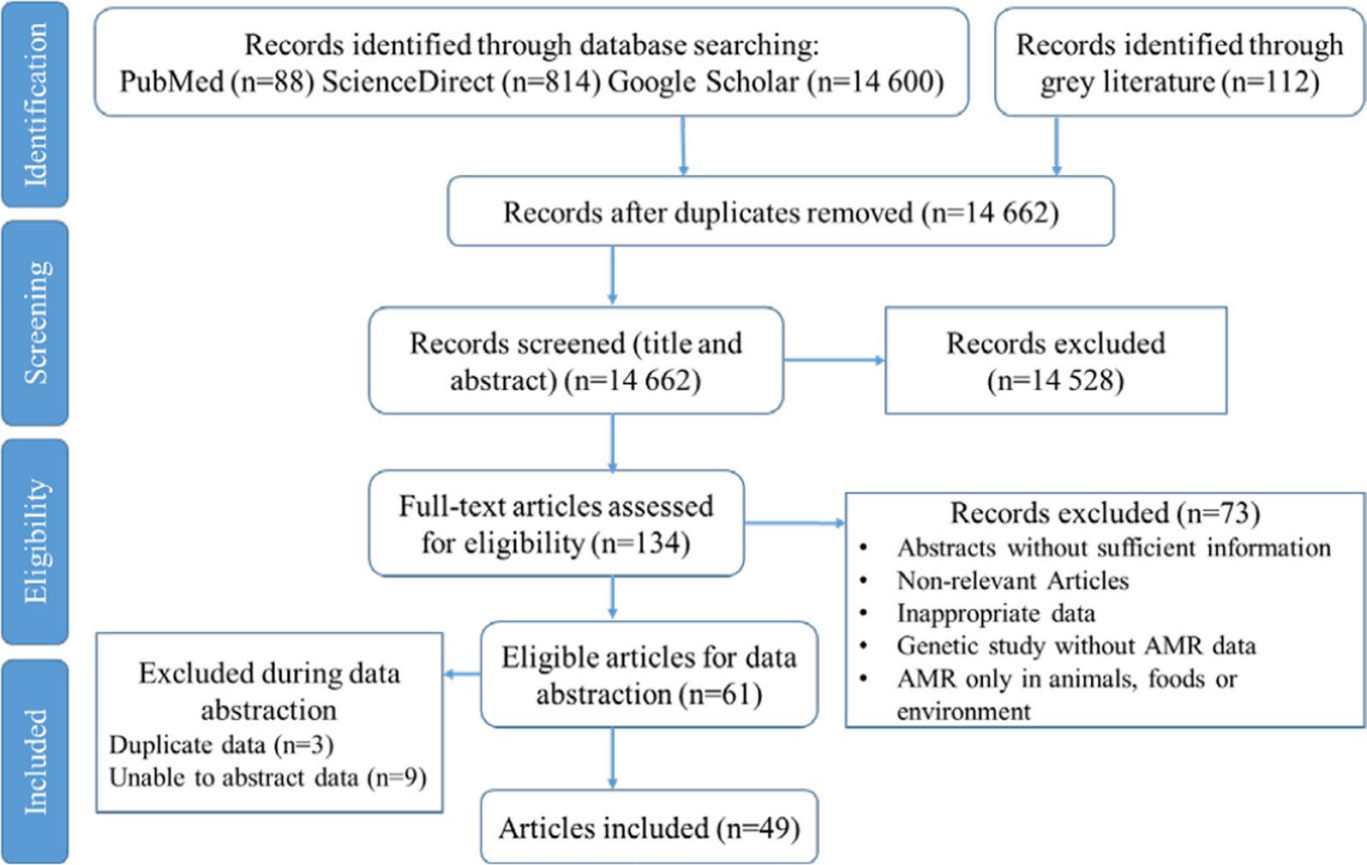
Flow diagram of the search results and selection of the included studies

PubMed, SciencDirect, and the Google Scholar were searched for papers from January 1, 2011 to December 20, 2021. Search strategy in PubMed database was performed on MeSH terms (see Additional file 2: Appendix 2). In addition, we researched related reviews and references for relevant studies. The design of this proposed scoping review methodology was informed by Arksey and O’Malley’s framework [33] and The Joanna Briggs Institute Reviewers’ Guidance [34]. The selection of articles for review is done by three-stage method whereby the title alone was examined, followed by looking at the abstract, and then examining the whole article (Fig. 1).

### Eligibility: inclusion and exclusion criteria

All original articles written in English or French languages reporting the prevalence of antibiotic resistance in bacteria strains isolated from humans by standard laboratory tests are included.

The inclusion criteria include:

- Reports on AMR in humans from Morocco,
- Information about antibiotic resistance of at least one bacterium,
- The denominator as total isolates clearly described for population-based studies,
- Correspondence and abstracts published with sufficient information on methodology and results.

The exclusion criteria include reports published before 2011, studies only focused on HIV or tuberculosis without AMR information, reviews, and studies without information on total studied isolates.

### Article quality assessment

The quality of each article is assessed using the modified critical appraisal checklist recommended by the Joanna Briggs Institute [35] (Additional file 2: Appendix 3). Quality assessment of studies was performed by two reviewers independently. Disagreements were resolved by a consensus-based discussion. Nine items are used as quality criteria for assessing the design, details of sample collection, processing and reporting on AMR methodologies.

### Data extraction and analysis

Data extraction is done using a predesigned and pretested database, developed for the purpose of this review using Microsoft Excel 2016 spreadsheet (Additional file 2: Appendix 4). Data extracted are name of first author, publication date, sample size, time and location of study, laboratory methodological information (pathogen identification and antimicrobial susceptibility testing methodology) and antibacterial resistance data.

Intermediate susceptibility, where reported, is considered as resistant. Where susceptibility rates are reported, without resistance rates, the resistance rates are calculated as the inverse of the susceptibility rates. Two authors independently collected data.

## Results

### Data and study characteristics

In total, 14,662 articles are collected from the initial literature search, and from them only 61 are eligible for data abstraction (Fig. 1). However, after full assessment, 12 articles are excluded due to data overlapping or duplication [36–38] and for difficulties to abstract data [39–47]. Finally, 49 papers fulfilling the inclusion criteria are included in the final analysis. Characteristics of included studies are summarized in Table 1.

**Table 1:** Characterization of included studies

Study	Study design	Time enrolled	Moroccan location	Isolate source and sample	Susceptibility method used	GLASS Organism(s) included
[110]	Prospective	2008–2009	Primary health care in Marrakech	660 nasopharyngeal samples (children under 2 years)	E-test	*S. pneumoniae*
[62]	Prospective	2008	Ibn Sina UH, Rabat	Nasal carriage of 54 hemodialyzed patients	DDM	*S. aureus*
[111]	Retrospective	Mar 2006–Jun 2010	Military Hospital Mohammed V, Rabat	307 samples corresponding to isolate bacterial strains from burn patients	DDM	*K. pneumoniae, E coli*
[112]	Survey	1998–2008	Ibn Rochd UH of Casablanca	955 consecutive non-duplicate isolates recovered at the microbiology laboratory	DDM	*S. pneumoniae*
[113]	Prospective	Jan 2010–Jan 2010	Private laboratory and Mohammed V Hospital Meknes	Urine samples from 480 inpatients and outpatients	DDM	*E. coli, Klebsiella.sp*
[114]	Surveillance	Sep 2007–Aug 2008	Ibn Rochd UH of Casablanca	185 children aged ≤ 5 years diagnosed with bacterial invasive infection	ADM and DDM	*S. Pneumoniae*
[115]	Prospective	July 2009–Dec 2010	UH Mohamed VI of Marrakesh	376 urine cytobacteriology (hospitalized infants)	DDM	*E. coli, K. pneumoniae*
[63]	Prospective	Jan–Jul 2007	Ibn Rochd UH of Casablanca	160 isolates from pathological samples of patients (79 cases) and nasal swabs (81) of cases and controls	DDM	*S. aureus*
[116]	Prospective	Jan 2010 -Dec 2011	Casablanca, El Jadida, Settat, Rabat, Meknes, Fez	1174 community acquired uropathogenic *E. coli* isolates	DDM	*E. Coli*
[59]	Prospective	Jan–Dec 2010	Ibn Sina University Hospital, Rabat	47 nonduplicate *A. baumannii* isolated from in-patients	DDM	*A. baumannii*
[117]	Prospective	Jan – Sep 2009	Military Hospital Mohammed V, Rabat	Non-redundant isolates of *P. aeruginosa* and *A. baumannii* from various samples	DDM	*A. baumannii*
[118]	Prospective	2010	UH Mohammed VI, Marrakech	38 cases of peritonitis (peritoneal fluid)	DDM	*E. coli, K. pneumoniae Streptococcus* spp.
[49]	Retrospective	Jul–Dec 2009	Five cities situated in north, central, west and south Morocco	171 men complaining of urethral discharge	E-Test	*N. gonorrhoeae*
[119]	Retrospective	May 2007–May 2009	UH Mohammed VI, Marrakech and several health centers	Nasopharyngeal samples taken from healthy children aged 1–24 months (660 children)	DDM	*S. pneumoniae*
[64]	Cross-sectional	Jan to Jun 2012	Hemodialysis centers in Fez region	Nasal swab specimens (143 hemodialyzed outpatients and 32 medical staff)	DDM	*S.aureus*
[120]	Retrospective	Mar to Jun 2012	Clinical laboratories in Tangier-Tetouan region	111 Enterobacteriaceae isolates were collected with patient information	ADM	*Enterobacteriaceae*
[121]	Prospective	Jan 2011–Jan 2012	CHU Ibn Sina Rabat	50 *A. baumannii* isolates from patients in intensive care unit	E-Test	*A. baumannii*
[122]	Prospective	Feb 2012–Mar 2013	CHU Ibn Sina Rabat	Ear swabs were collected from patients at the pediatric hospital	DDM	*E. coli, K. pneumoniae, Streptococcus* spp*.*
[65]	Prospective	Nov 2008–Feb 2009	Private hemodialysis centres in Casablanca	Nasal swabs (145 patients and 42 personnel)	DDM	*S. aureus*
[123]	Prospective	Jun–Aug 2011	Ibn Rochd UH of Casablanca	166 isolates recovered from urine (n = 80), pus (n = 34), blood (n = 24), sputum (n = 11) and others (n = 17)	DDM	*K. pneumoniae*
[124]	Retrospective	Apr 2012–July 2013	Military Hospital Mohammed V, Rabat	Isolates from inpatients and outpatients. Urine isolates represented 82%	DDM	*E. coli*
[125]	Prospective	Jan 2012–Dec 2013	UH Mohamed VI of Marrakesh	406 enterobacteriaceae strains isolated (urinary samples of hospitalized children)	DDM	*E. coli, Klebsiella.sp*
[126]	Retrospective	Nov 2010–Dec 2012	Mohammed V Hospital Meknes	150 patients infected by *E. coli* from hematology (68.7%), urology (22%) and burn (16%) wards	DDM	*E. coli*
[127]	Retrospective	Jan 2010–Dec 2012	Avicenne Teaching Hospital, Marrakech	*K. pneumoniae* strains (urine samples)	BMD	*K. pneumoniae*
[128]	Retrospective	2010–2012	Avicenne Teaching Hospital, Marrakech	Uropathogenic *E. coli* (urine samples from in and outpatients	BMD	*E. coli*
[129]	Prospective	2007–2014	Ibn Rochd University Hospital Casablanca	655 *S. pneumoniae* isolates (pediatric and adult patients)	DDM	*S. pneumoniae*
[130]	Retrospective	Jan 2012–Dec 2013	Abderrahim El Harrouchi children hospital of Casablanca	34 patients (newborns)	ADM	*E. Coli, K. pneumoniae*
[48]	Not Mentioned	Mar 2001–Mar 2012	Pediatric hospital of Rabat	Children under-5 with acute moderate-to-severe diarrhea	DDM	*Salmonella* spp*., E. coli, Shigella* spp.
[57]	Retrospective	2012–2014	Military Hospital Mohammed V, Rabat	clinical isolates of *Acinetobacter sp* (samples from inpatients)	DDM	*A. baumannii*
[52]	Prospective	Dec 2012–Nov 2013	Military Hospital Mohammed V, Rabat	46 episodes of bacteremia recorded in 39 patients	DDM	*A. baumannii, K. pneumonia*
[131]	Prospective	Feb–July 2013	UH Hassan II, Fez	Hospitalized neonates (rectal swab specimens)	DDM	*E. coli, K. pneumonia*
[51]	Prospective	2012–2015	Laboratories of medical analysis from North-West of Morocco	516 clinical isolates. Specimens including urine (485), pus (15), vaginal specimen (10) and other ones with low rate	DDM and BMD	*E. coli, Klebsiella* spp.
[56]	Prospective	2010–2014	Ibn Rochd University Hospital Casablanca	4232 non-duplicate blood cultures	DDM	*A. baumannii*
[50]	Prospective	2003–2009	Pasteur Ins., Casablanca	26 isolates from foodstuffs and humans samples	ADM	*Salmonella Infantis*
[66]	Retrospective	Apr 2007–Dec 2015	Military Hospital Mohammed V, Rabat	451 wounds, 126 blood cultures and 50 catheter samples from burn inpatients	DDM	*E. coli, K. pneumoniae, Streptococcus* spp.
[132]	Prospective	Feb 2013–July 2015	UH Hassan II, Fez	Intestinal carriage (newborns hospitalized in neonatal intensive care unit)	DDM	*A. baumannii*
[60]	Prospective	Apr 2015–Jul 2016	MH, Regional hospital of Meknes and UH Center of Casablanca	Specimens including urine, pus, distal bronchial Levy protected, bronchial aspirate, central catheter, blood cultures and others	ADM	*A. baumannii*
[133]	Retrospective	Jan 2013–Dec 2017	UH Mohamed VI of Marrakesh	4 769 cytobacteriological examinations of urine (hospitalized children 0–17 years)	DDM	*E. Coli*
[61]	Prospective	Jun 2017–Jun 2018	UH Mohamed VI of Marrakesh	Nasal carriage of *S. aureus* in 300 children consulting at different pediatric specialties	ADM and DDM	*S. aureus*
[53]	Retrospective	Jun 2015–Jun 2016	Mohammed V Hospital Meknes	126 burn patients (86 infected)	DDM	*E. Coli*
[55]	Case control	Mar 2015–Mar 2016	UH Mohamed VI of Marrakesh	479 patients from the clinical and surgical ICU enrolled with a first clinical episode of HAI	DDM	*A. baumannii*
[134]	Survey	2015–2018	UH Ibn Rochd, Casablanca	19 *S. pneumoniae* isolates (community patients with respiratory tract infections)	BMD	*S. pneumoniae*
[67]	Case report	2017	UH Ibn Rochd, Casablanca	35-year-old female patient. Swab sample from the surgical wound	DDM	*S. pneumoniae*
[135]	Prospective	Feb 2013–July 2015	UH Hassan II, Fez	Intestinal carriage (newborns hospitalized in neonatal intensive care unit)	DDM	*E. coli, K. pneumoniae*
[136]	Retrospective	Jan 2016–Jun 2019	Cheikh Khalifa International UH, Casablanca	Isolates of ESBL-EC isolated from 670 urine samples	DDM	*E. coli*
[137]	Prospective	Jan 2017–Dec 2018	Medical analysis laboratories in Casablanca city	2090 urines samples collected throughout Casablanca from the health facilities	DDM	*E. coli*
[58]	Retrospective	Jun 2016–Dec 2018	UH Mohammed VI of Oujda	863 positive blood cultures in the microbiology laboratory (adults, children and newborns)	Not indicated	*A. baumannii*
[138]	Retrospective	Jan 2012–Dec 2018	Military Hospital Mohammed V, Rabat	10,324 isolates of *E. coli*	DDM	*E. coli*
[54]	Case control	Jan–Dec 2018	UH Mohammed VI of Marrakesh	131 non-duplicate carbapenem-resistant Enterobacteriaceae	DDM	*E. coli, K. pneumoniae*

UH, University hospital; ADM, agar dilution method; DDM, disk diffusion method; BMD, broth microdilution

Of the 49 included studies, 13 reported isolates from children only, while 14 not reported age of patients. The majority of included studies [38] used the disk diffusion method as the antibiotic-susceptibility test. Some studies used agar dilution and broth dilution combined, referred to as MIC testing for the analysis. The most commonly reported organism was *E. coli*, with AMR data reported by 22 papers. In contrast, AMR data is reported by one paper for *Shigella* spp*.* [48], one paper for *N. gonorrhoea* [49] and two papers for *Salmonella* spp*.* [48, 50] (Table 1).

### Microbial resistance patterns

#### Escherichia coli

**Fig. 2:**
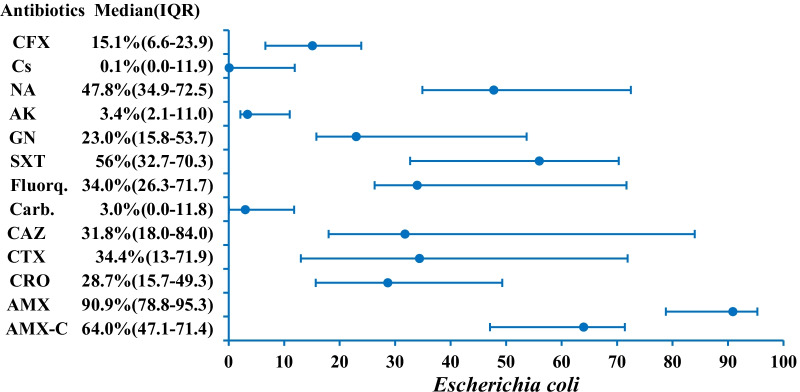
AMR profile of *E. coli* in the form of median resistance with interquartile range. *AK* Amikacin, *AMX-C* Amoxicillin-clavulanic acid, *AMX* amoxicillin, *Carb* Carbapenems, *CRO* Ceftriaxone, *CTX* Cefotaxime, *CAZ* Ceftazidime, *CFX* Cefoxitin, *Cs* Colistin, *Fluorq* Fluoroquinolones, *GN* Gentamicin, *NA* Nalidixic acid, *SXT* Trimethoprim-sulfamethoxazole

The most commonly reported bacterium was *E.coli*. it is reported in 22 studies (Table 1). Median resistances are calculated as 64.0% (n = 21, IQR 47.1–71.4), 90.9% (n = 13, IQR 78.8–95.3), 34.0% (n = 23, IQR 26.3–71.7), 56.0% (n = 19, IQR 32.7–70.3), 23.0% (n = 23, IQR 15.8–53.7), 3.4% (n = 22, IQR 2.1–11.0), 47.8% (n = 9, IQR 34.9–72.5), and 15.1% (n = 11, IQR 6.6–23.9) for amoxicillin-clavulanic acid, amoxicillin, fluoroquinolones, co-trimoxazole, gentamicin, amikacin, nalidixic acid and cefoxitin, respectively (Fig. 2; Additional file 1: Table S1). For 3GC, median resistances are calculated as 28.7% (n = 8, IQR 15.7–49.3), 34.4% (n = 14, IQR 13.0–71.9), and 31.8% (n = 12, IQR 18.0–84.0) for ceftriaxone, cefotaxime and ceftazidime, respectively. Colistin resistance is reported as 0.1% (n = 7, IQR 0.0–11.9). Carbapenem resistance is studied in 21 papers and calculated as 3.0% (IQR 0.0–11.8).

#### Klebsiella pneumonia

**Fig. 3:**
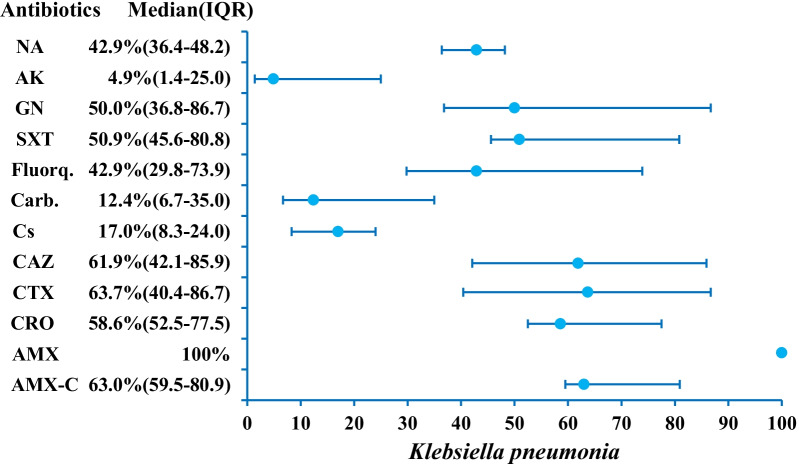
AMR profile of *K. pneumonia* in the form of median resistance with interquartile range. *AK* Amikacin, *AMX-C* Amoxicillin-clavulanic acid, *AMX* amoxicillin, *Carb* Carbapenems, *CRO* Ceftriaxone, *CTX* Cefotaxime, *CAZ* Ceftazidime, *CFX* Cefoxitin, *Cs* Colistin, *Fluorq* Fluoroquinolones, *GN* Gentamicin, *NA* Nalidixic acid, *SXT* Trimethoprim-sulfamethoxazole

AMR data on *K. pneumonia* is reported in 16 studies (Table 1). Median resistances are calculated as 63.0% (n = 15, IQR 59.5–80.9), 100.0% (n = 7), 42.9% (n = 15, IQR 29.8–73.9), 50.9% (n = 12, IQR 45.6–80.8), 50.0% (n = 15, IQR 36.8–86.7), 4.9% (n = 14, IQR 1.4–25.0), 42.9% (n = 5, IQR 36.4–48.2) for amoxicillin-clavulanic acid, amoxicillin, fluoroquinolones, co-trimoxazole, gentamicin, amikacin and nalidixic acid respectively (Fig. 3; Additional file 1: Table S2). Carbapenem resistance is reported by 15 papers with a median rate of 12.4% (IQR 6.7–35.0). For 3GC, median resistances are calculated as 58.6% (n = 6, IQR 52.5–77.5), 63.7% (n = 9, IQR 40.4–86.7), 61.9% (n = 10, IQR 42.1–85.9) for ceftriaxone, cefotaxime and ceftazidime respectively. Colistin resistance is reported as 17.0% (IQR 8.3–24.0) in four studies [51–54].

#### Acinetobacter baumannii

**Fig. 4:**
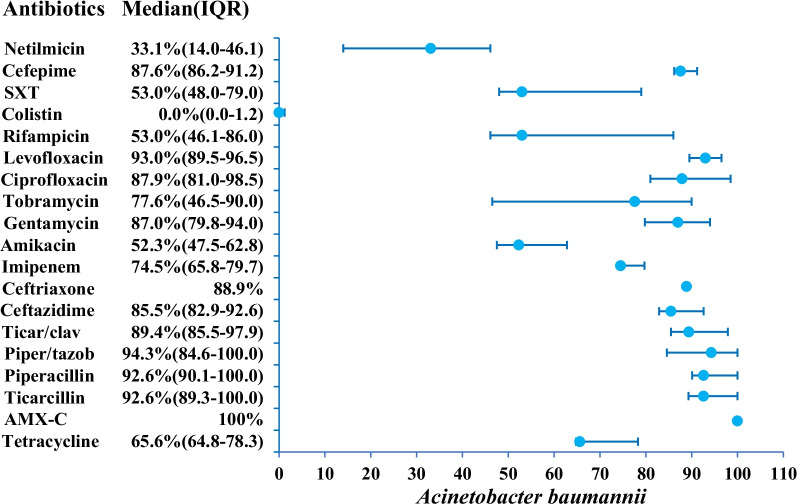
AMR profile of *A. baumannii* in the form of median resistance with interquartile range. *AMX-C* Amoxicillin-clavulanic acid, *SXT* Trimethoprim-sulfamethoxazole

Thirteen papers reported data for *A. baumannii* (Table 1). Except for El Mekes et al. study [55], all papers reported imipenem resistance with a median 74.5% (IQR 65.8–79.7). Three studies reported resistance rates of 90.9%, 64.0% and 65.6% to tetracyclines [56–58]. Higher resistance to ticarcillin and piperacillin is reported in nine studies (92.6%, IQR 89.3–100.0) (Fig. 4; Additional file 1: Table S3). AMR resistance to 3GC, especially represented by ceftazidime, was reported as a median of 85.5% (n = 10, IQR 82.9–92.6). Gentamicin and amikacin resistance was reported with rates of 87.0% (n = 9, IQR 79.8–94.0) and 52.3% (n = 11, IQR 47.5–62.8), respectively. Colistin resistance is reported in eight studies as 0.0%, (IQR 0.0–1.2). Cefepime (4CG) resistance is reported by four studies as 87.6% (IQR 86.2–91.2) [57–60].

#### *Salmonella* spp.

Two papers report resistance data for *Salmonella* spp*.* in humans [48, 50]. In the study of Benmessaoud et al*.* [48], resistance to 3CG, 4CG, imipenem and amikacin is not detected. Resistance to tetracyclines, fluoroquinolones (ciprofloxacin and levofloxacin) and co-trimoxazole is reported as 60.0%, 20.0% and 40.0%, respectively. The results reported by Ed-Dra et al*.* [50] show that 84.6% (22/26) of the *Salmonella infantis* strains were susceptible to all of the 14 antibiotics tested. Three strains are resistant to tetracycline, two strains had low-level β-lactam resistance and one strain is resistant to streptomycin and sulfonamide.

#### *Shigella* spp.

One study reports AMR data for *Shigella* spp*.* among nine isolates including six *S. sonnei* [48]. No resistance found to 3CG, fluoroquinolones and imipenem. Resistance higher than 50% is reported to tetracycline (55.5%) and co-trimoxazole (66.7% for all strains and 83.3% for *S. sonnei*).

#### Neisseria gonorrhoeae

One study reports AMR data for *N. gonorrhoeae* among 72 isolates recruited from 171 men [49]. Resistance to ciprofloxacin is identified in 86.8% of *N. gonorrhoeae* strains, 16.2% are resistant to penicillin and 92.6% were resistant to tetracycline. All the isolates are 100% susceptible to ceftriaxone, cefixime and spectinomycin. In this study, evolution of resistance in *N. gonorrhoeae* strains isolated in 2001 and 2009 was reported. The AMR study in 2009 demonstrated an increasing trend of resistance in *N. gonorrhoeae* to tetracycline (from 59.7% in 2001 to 92.6% in 2009) and to ciprofloxacin (from 2.6% in 2001 to 86.7% in 2009).

#### Staphylococcus aureus

Six papers report *S. aureus* among human populations [61–66]. MRSA rates range from 1.6% to 31.1%. Diawara et al*.* [64] report that only one strain per 62 isolates (1.6%) expressed an inhibition around cefoxitin and moxalactam disks, which is confirmed as MRSA. In the study of Ed-dyb et al*.* [61], 49 strains of *S. aureus* are isolated and the prevalence of MRSA is 4% (2/49) of *S. aureus* isolates. The rate of MRSA in hemodialyzed patients is 2.1% (1/47) in the study of Elazhari et al*.* [65]. In the study of Frikh et al*.* [66], *S. aureus* is the second most prevalent isolate with a rate of 14.9%, of which 31.1% are MRSA. The prevalence of MRSA strains is 12.5% (3/24) in the study of Souly et al*.* [62]. The overall prevalence of MRSA in the study of Zrouil et al*.* [63] is 18.4%.

#### Streptococcus pneumoniae

**Fig. 5:**
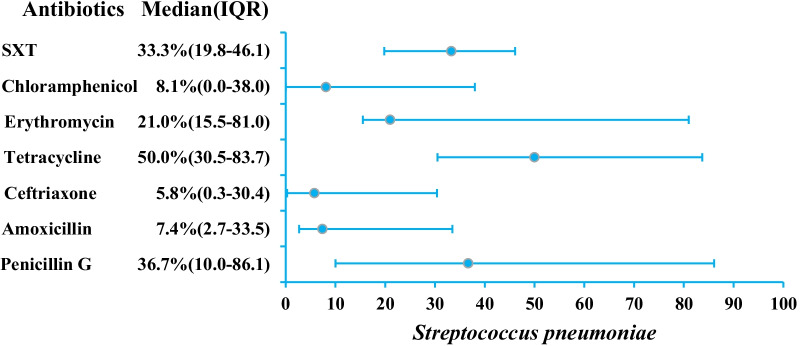
AMR profile of *St. pneumonie* in the form of median resistance with interquartile range. *SXT* Trimethoprim-sulfamethoxazole

AMR data for *St. pneumonie* is reported by seven studies and does not exceed 50.0% as a median (resistance to tertracycline with IQR 30.5–83.7) (Table 1; Fig. 5; Additional file 1: Table S4). Resistance to penicillin G, co-trimoxazole, erythromycin is reported as 36.7% (n = 6, IQR 10.0–86.1), 33.3% (n = 5, IQR 19.8–46.1) and 21.0% (n = 5, IQR 15.5–81.0), respectively. Ceftriaxone resistance is reported by four studies as a median of 5.8% (IQR 0.3–30.4).

In a case study [67], Néhémie et al. reported characteristics of a 35-year-old female patient. An ovarian transposition is performed in the Ibn Rochd University Hospital Centre of Casablanca. Antibiotic susceptibility tests are performed by disc diffusion and E-test method. The strain isolated is resistant to oxacillin, erythromycin, ampicillin, clindamycin, penicillin G and co-trimoxazole. It is only susceptible to vancomycin, levofloxacin and chloramphenicol and intermediate to ceftriaxone.

## Discussion

Over the last decade in Morocco, there has been no comprehensive review dealing with the AMR prevalence using the global antimicrobial resistance surveillance system (GLASS). This attempt seeks, hopefully, to fill the gap and clarify the AMR status in the country’s regions. The AMR data depicts high heterogeneity due to unstandardized laboratory methods, clinical conditions, and a few isolates. This makes drawing firm conclusions highly challenging. However, resistance rates to several key clinically important antibiotics are found to be alarmingly high.

To this end, the European Committee on Antimicrobial Susceptibility Testing (EUCAST) standards are recommended over the Clinical and Laboratory Standards Institute (CLSI) guidelines. Moreover, improved access to quality assurance is needed to enhance the current WHO initiative, and scale up the global antimicrobial surveillance system (GLASS) based on country-specific priority pathogens [9].

In a recent systematic review conducted in the MENA region [68], it is shown that the lack of consistency and harmonization in the regional surveillance system is not a prerogative of the Middle East, as is the case in developed countries.

The most frequently GLASS pathogens belong to the Enterobacteriaceae family (*E. coli* and *K. pneumoniae*), *A. baumannii*, and *S. aureus*. They have been described in most of selected papers. Other systematic reviews, conducted on AMR in the Middle East [68] and Africa [69], have reported the same results. Concerning *Shigella* spp. and *N. gonorrhoeae*, each has been cited by only one paper. *Shigella* spp. is the second leading cause of diarrheal mortality, which accounts for 13.2% of diarrheal deaths globally [70] whereas, *N. gonorrhoeae* causes high levels of morbidity in LMICs, and shows the rapid development of AMR [8, 9].

Enterobacterales are a large order of different types of bacteria that commonly cause infections both in healthcare settings and communities. This family represented especially by *E. coli* and *K. pneumoniae* can produce extended-spectrum beta-lactamases (ESBLs) Enzymes. The latter break down some commonly used antibiotics such as penicillins and cephalosporins, which render them inefficient [71]. The WHO has recently published a global priority list of antibiotic-resistant bacteria, which includes ESBL-producing *Enterobacteriaceae* and carbapenemase-producing *Enterobacteriaceae* [8, 9]. Carbapenem belong to the category of β-lactams, which has a broader spectrum of activity. It bind to the bacterial cell wall and inhibits growth. It also results in damage to the cell wall, which frequently leads to cell lysis and death [13, 72, 73]. Carbapenem resistance may be caused by different mechanisms, one of them being inducible overexpression of chromosomal cephalosporinases combined with porin loss [74, 75]. *Enterobacteriaceae* with ESBL/carbapenemase genes are bestowed with highly multi-drug resistance among humans, animals, and food chains [76]. Moreover, careless use of these antibiotic classes would co-select for resistance genotypes against the others [76].

The proportion of AMR driven from this review is alarming. The highest proportion of studies on both *E. coli* and *K. pneumoniae* are related to UTIs. Such cases require more complex treatments [9]. Such infections might require hospitalization and intravenous injection of carbapenem antibiotics. In this review, the carbapenem-resistance proportion among GLASS *Enterobacteriaceae* appears like other reports from Africa [69] and the Middle East [68], but higher than those described in most European countries [77]. In this context, the prevalence of carbapenemase-producing *K. pneumoniae* and *E. coli*, per 10,000 hospital admissions, presents an average of 1.3 (6.0 in Italy, 0.02 in Norway). The incidence per 100,000 hospital patient-days ranged from 17.3 in Greece to 0.09 in Lithuania, with a mean of 2.5 across all the countries. In China, the overall carbapenem-resistant *Enterobacteriaceae* infection incidence per 10,000 discharges was 4.0 and varies significantly by region [78]. However, no carbapenem-resistant *Enterobacteriaceae* is found in a recent systematic review from Cambodia [79]. Carbapenemases have a global distribution, but substantial variability exists at the regional and continental levels.

Recently, different products are under evaluation and over thirty antibiotics are active against the most dangerous pathogens included in the WHO’s priority pathogens [80, 81]. Many of them consist of combinations of new β-lactams and β-lactam inhibitors. d-mannose derivatives and glycomimetics are reported as a promising, valuable, effective, feasible and cost-effective way to treat UTIs especially, urgent clinical trials [82, 83].

In the past decade, numerous review papers have highlighted the rising problem of colistin resistance worldwide, especially with *E. coli, K. pneumonia*, and *A. Baumanii* in the human community [16, 68, 69, 84–87]. Current and emerging colistin resistance may be explained by its high usage in the animal field, and this not only as an infection-healing drug but also as a growth promoter and protective agent [88]. Following this study, several reviews have also reported high 3GC, co-trimoxazole, fluoroquinolones, and gentamicin resistance among *E. coli* and *K. pneumoniae* isolates [68, 69, 87].

In the current review, the pathogens isolated from humans such as *Salmonella* spp., *Shigella* spp*.*, and *N. gonorrhoeae* are understudied in the Morocco context. However, AMR in *Salmonella* spp. from foods and environmental sources is mentioned by several studies [89, 90]. Such finding is also revealed by other systematic reviews in other countries [68, 69]. On the other hand, *N. gonorrhoeae* is known for its high resistance to ciprofloxacin [91, 92]. Of note, ciprofloxacin, which is used to treat gonococcal infections, done by, was replaced by ceftriaxone in the Moroccan context [49]. This decision is sustained by previous studies [93] stating that penicillin, tetracycline, and ciprofloxacin should not be used for *N. gonorrhoeae* management in Morocco. For *Salmonella* spp., the prevalence of fluoroquinolone resistance has exceeded 30% in many areas of the Arab World [94]. This remains significantly high when compared with the Moroccan context, where it does not surpass 20%. As recommended by Ranjbar et al*.* [28] a clear virulence gene profile of *Shigella* may lead to have an accurate diagnosis and a definite treatment relating to different pathogenic strains. In a recent study on *Shigella* in Morocco, the dual contribution of SfGtr4 and SfPgdA genes to the pathogenicity and the regulation biofilm formation by *S. flexneri* is demonstrated [95].

The epidemiology of *S. aureus*, especially that of MRSA, has shown a rapid evolution over the last years. Global surveillance has emphasized that MRSA represents a problem in all countries showing an increase in the mortality and need to use last-resource antibiotics [8, 9, 96]. The proportion of MRSA (30%) reported in this review is still higher than that mentioned in the European countries (16.9%) [97], but lower than those reported in Asia (28–70%) [98], and Africa (53%) [99]. While the treatment options for MRSA are still limited, there are several new antimicrobials under development [100]. *S. pneumoniae* is reported as a major cause of community-acquired pneumonia, meningitis, sepsis, bacteremia, and otitis media [101, 102]. A decline in susceptibility of *S. pneumoniae* to commonly used beta-lactams, fluoroquinolones, and macrolides is mentioned by several studies [101, 103, 104].

Although the findings of this study may seem useful, some limitations must be considered when the interpretation of the results is required. The strict focus on GLASS bacteria might have led to oversight of important pathogens like *Helicobacter pylori* [105, 106], and *Pseudomonas aeruginosa* [107, 108], which are of significate public health concern in AMR. The Validity and generalizability of the findings to the entire country’s regions might be affected by the clinical-based, cross-sectional study design of the published papers, mainly collected from Casablanca and Marrakech cities. Besides, there is high variability among the criteria relevant to methodology and interpretation. This is consonant with the data depicted elsewhere in recent similar reviews [68, 69, 79, 87]. There are some calls to adopt standardized AMR data presented in published papers, wishing to make the findings interpretable and comparable from the perspective of scarce homogeneity [109]. Despite these limitations, the high proportion of AMR detected in this review has a certain degree of validity.

## Conclusions

In summary, this review highlights that data on AMR in Morocco are limited but improving. Overall, there are significant similarities in AMR tendency in comparison with other countries worldwide. The recent joining of Morocco to the GLASS system will improve the accuracy, quality, and comparability of data collected on AMR.

## Supplementary Information

**Additional file 1:** **Table S1**. Resistance rates for *E. coli* isolates. **Table S2**. Resistance rates for *K. pneumonia *isolates. **Table S3**. Resistance rates for *A. baumannii *isolates. **Table S4**. Resistance rates for *S. pneumonia *isolates.

**Additional file 2: Appendix 1**. GLASS specific antibiotic susceptibility testing against individual organisms (WHO, 2020). **Appendix 2**. Search strategy. **Appendix 3**. Joanna Briggs Institute’s critical appraisal checklist for studies reporting prevalence data. **Appendix 4**. Data extraction form

## Data Availability

All data generated or analyzed during this study are included in this published article and its Additional files.

## Abbreviations

ABRI: *Acinetobacter baumannii* resistant to imipenem
ADM: Agar dilution method
AK: Amikacin
AMR: Antimicrobial resistance
AMX-C: Amoxicillin-clavulanic acid
AMX: Amoxicillin
BMD: Broth microdilution
C3G: Third-generation cephalosporins
Carb: Carbapenems
CAZ: Ceftazidime
CFX: Cefoxitin
CIP: Ciprofloxacin
CLSI: Clinical and Laboratory Standards Institute
CRO: Ceftriaxone
Cs: Colistin
CTX: Cefotaxime
DDM: Disk diffusion method
ESBL: Extended spectrum beta lactamase
E Test: Epsilometer test
*E. coli*: *Escherichia coli*
EUCAST: European Committee on Antimicrobial Susceptibility Testing
GLASS: Global antimicrobial resistance surveillance system
GM: Gentamicin
Fluorq: Fluoroquinolones
HIV: Human immunodeficiency virus
MENA: Middle East and North Africa
IQR: Interquartile range
MIC: Minimum inhibitory concentration
NA: Nalidixic acid
MRSA: Methicillin-resistant *Staphylococcus aureus*
SXT: Trimethoprim-sulfamethoxazole
UH: University Hospital
WHO: World Health Organization
