# Supplementary material for: Antimicrobial resistance among GLASS pathogens in Morocco: an epidemiological scoping review
Source: BMC Infect Dis. 2022 May 7;22:438. doi: 10.1186/s12879-022-07412-4 (PMC9077917; doi:10.1186/s12879-022-07412-4)
Supplement: Supplementary file 1 — Additional file 1: Table S1. Resistance rates for E. coli isolates. Table S2. Resistance rates for K. pneumonia isolates. Table S3. Resistance rates for A. baumannii isolates. Table S4. Resistance rates for S. pneumonia isolates. [file 12879_2022_7412_MOESM1_ESM.docx]

**Table S1.** Resistance rates for *E. coli* isolates.

| **Study** | **Nb of isolate** | **AMX-C**  **n(%)** | **AMX**  **n(%)** | **CRO**  **n(%)** | **CTX**  **n(%)** | **CAZ**  **n(%)** | **Carb.**  **n(%)** | **Fluorq.**  **n(%)** | **SXT**  **n(%)** | **GN**  **n(%)** | **AK**  **n(%)** | **NA**  **n(%)** | **Cs**  **n(%)** | **CFX**  **n(%)** |
| --- | --- | --- | --- | --- | --- | --- | --- | --- | --- | --- | --- | --- | --- | --- |
| [111] | 7 | 5(71.4) | - | - | 0(0.0) | - | 0(0.0) | 0(0.0) | 2(28.6) | - | 0(0.0) | - | - | - |
| [115] | 133 | 92(69.0) | - | - | 7(5.3) | - | 0(0.0) | 16(12.0) | 70(52.5) | 12(9.0) | 3(2.0) | - | - | - |
| [113] | 844 | 528(62.5) | - | - | - | 148(17.5) | 59(7.0) | 253(30.0) | 506(60.0) | 224(26.5) | 72(8.5) | - | - | - |
| [118] | 15 | 10(64.0) | - | 14(93.0)¥ | - | - | 15(100.0) | - | - | 15(100.0) | 15(100.0) | - | - | - |
| [116] | 49 | 49(100.0) | 49(100.0) | - | 49(100.0) | 49(100.0) | 5(10.2) | 40(81.6) | 38(77.6) | 31(63.3) | 10(20.4) | 40(81.6) | - | 19(38.8) |
| [120] | 69 | 40(58.0) | 45(65.2) | 30(43.5) | 24(34.8) | 22(31.9) | 0(0.0) | 26(37.7) | - | 9(13.0) | 0(0.0) | 33(47.8) | - | - |
| [122] | 3 | 1(33.3) | 3(100.0) | 2(66.6) | - | - | - | 2(66.6) | - | 0(0.0) | - | - | - | - |
| [124] | * | 532/1263 (42.1) | - | 60/1032 (5.8) | - | - | 31/1251 (2.5) | 259/1020 (28.9) | 456/1244 (36.7) | 129/1137 (11.3) | 18/1250 (1.4) | - | - | 56/1256 (4.4) |
| [128] | 924 | 436(47.1) | 624(67.5) | - | - | - | 0(0.0) | 243(26.4) | 522(56.5) | 164(17.8) | 106(11.5) | - | - | - |
| [125] | 224 | 148(66.0) | 184(82.0) | 47(21.0)¥ | - | - | 0(0.0) | 27(12.0) | 105(47.0) | 38(17.0) | 2(1.0) | - | - | - |
| [126] † | 690 | 296(48.6) | 480(78.8) | - | 69(11.3) | 83(13.6) | 0(0.0) | 259(42.5) | 141(23.2) | 159(26.1) | 14(2.3) | 259(42.5) | 0(0.0) | 92(15.1) |
| [126] ‡ | 150 | 130(86.7) | 143(95.3) | - | 53(35.3) | 45(30.0) | 0(0.0) | 114(76.0) | 141(94.0) | 26(17.3) | 14(9.3) | 114(76.0) | 0(0.0) | 13(8.7) |
| [48] | 71 | 36(50.7) | - | - | 2(2.8) | 4(5.6) | 1(1.4) | 2(2.8) | 40(56.3) | 11(15.5) | 2(2.8) | 7(9.8) | - | 0(0.0) |
| [131] | 67 | 58(86.6) | 61(91.0) | - | 56(83.6) | 56(83.6) | - | 44(65.7) | 13(19.4) | 57(85.1) | 6(9.0) | 44(65.7) | - | - |
| [51] | 398 | 257(64.6) | - | - | 96(24.1) | 126(31.7) | 23(5.8) | 104(26.1) | - | 85(21.4) | 10(2.5) | 138(34.7) | 45(11.3) | 96(24.1) |
| [133] | Not available | - | (80.0) | - | (34.0) | - | (2.0) | (15.4) | (56.0) | (16.0) | (2.7) | - | - | - |
| [53] | 11 | 11(100.0) | 10(90.9) | 4(36.4) | 2(18.2) | 2(18.2) | 2(18.2) | 8(72.7) | 7(63.6) | 4(36.4) | 2(18.2) | - | 1(12.5) | - |
| [55] | 25 | - | - | - | - | - | 10(41.0) | 20(81.0) | 20(81.0) | 18(71.0) | 4(16.0) | - | 0(0.0) | - |
| [135] | 152 on admission | 65(42.8) | 88(57,9) | - | 56(36.8) | 56(36.8) | 18(11.8) | 46(30.2) | 38(25.0) | 51(33.6) | 2(3.8) | 53(34.9) | - | 36(23.7) |
| [135] | 109 during NICU stay | 96(88.1) | 100(91,7) | - | 93(85.3) | 93(85.3) | 26(23.9) | 77(70.6) | 28(25.7) | 89(81.7) | 0(0.0) | 79(72.5) | - | 63(57.8) |
| [136] | * | 171(67) | 252(100) | - | 221(97.0) | 233(93.0) | 9(4.0) | 235(94.0) | 184(80.0) | 109(44.0) | 83(34.0) | - | 1(0.1) | 28(12.0) |
| [137] | 230 | 88(38.2) | - | 39(16.9)¥ | - | - | - | 62(27.0) | - | 53(23.0) | 16(7.0) | - | - | 39(17.0) |
| [138] | 10 324 | 3717(36.0) | - | 1239(12)¥ | - | - | 310(3.0) | 3510(34.0) | 4233(41.0) | 1033(10.0) | 310(3.0) | - | - | 310(3.0) |
| [54] | 13 | - | - | - | - | - | 13(100.0) | 11(84.6) | 10(76.9) | 13(100.0) | - | - | 5(38.5) | - |
| **Median (IQR)** |  | 64.0(47.1-71.4) | 90.9(78.8-95.3) | 28.7(15.7-49.3) | 34.4(13.0-71.9) | 31.8(18.0-84.0) | 3.0(0.0-11.8) | 34.0(26.3-71.7) | 56.0(32.7-70.3) | 23.0(15.8-53.7) | 3.4(2.1-11.0) | 47.8(34.9-72.5) | 0.1(0.0-11.9) | 15.1(6.6-23.9) |

† *E. coli* isolated from community setting; ‡ *E. coli* isolated from hospital setting; * number of isolates varied by antibiotic tested; ¥ for 3GC.

3GC, Third-generation cephalosporins (CRO, CTX and CAZ); AMX-C, Amoxicillin-clavulanic acid; AMX, amoxicillin; CRO, Ceftriaxone; CTX, Cefotaxime; CAZ, Ceftazidime; Carb, Carbapenems; Fluorq, Fluoroquinolones; SXT, Trimethoprim-sulfamethoxazole; GM, Gentamicin; AK, Amikacin; NA, Nalidixic acid; Cs, Colistin; CFX, Cefoxitin.

**Table S2.** Resistance rates for *K. pneumonia* isolates

| **Study** | **Nb of isolate** | **AMX-C**  **n(%)** | **AMX**  **n(%)** | **CRO n(%)** | **CTX**  **n(%)** | **CAZ**  **n(%)** | **Cs**  **n(%)** | **Carb.**  **n(%)** | **Fluorq.**  **n(%)** | **SXT**  **n(%)** | **GN**  **n(%)** | **AK**  **n(%)** | **NA**  **n(%)** |
| --- | --- | --- | --- | --- | --- | --- | --- | --- | --- | --- | --- | --- | --- |
| [111] | 18 | 7(39.3) | - | - | 7(39.0) | - | - | 1(5.5) | 5(28.0) | 8(44.4) | - | 3(16.7) | - |
| [115] | 32 | (42.0) | (100.0) | - | - | - | - | 0(0.0) | (14.0) | (46.0) | (21.0) | (5.0) | - |
| [113] | 384 | 243(63.0) | - | 92(24.0) | - | 148(39.0) | - | 4(1.0) | 142(37.0) | 196(51.0) | 133(35.0) | 273(71.0) | - |
| [118] | 5 | 3(60;0) | - | - | - | 5(100.0)* | - | 5(100.0) | - | - | 5(100.0) | 5(100.0) | - |
| [123] | 166 | 91(54.8) | - | - | 67(40.4) | 67(40.4) | - | 11(6.6) | 61(36.8) | 86(51.8) | 64(38.6) | 8(4.8) | - |
| [120] | 28 | 20(71.4) | - | 16(57.1) | 14(50.0) | 13(46.4) | - | 5(17.9) | 12(42.9) | - | 12(42.9) | 1(3.6) | 12(42.9) |
| [122] | 3 | 2(66.6) | 2(66.6) | 3(100.0) | - | - | - | - | 3(100.0) | - | 2(66.3) | - | - |
| [127] | 321 | 321(100.0) (51)¥ | - | - | - | - | - | 23(7.0) (0.0)¥ | 72(84.0) (32.0)¥ | 286(89.0) (61.0)¥ | 286(89.0) (21.0)¥ | 160(50.0) (11.0)¥ | - |
| [125] | 122 | 72(59.0) | 122(100.0) | 62(51.0)** | - | - | - | 4(3.0) | 31(25.0) | 57(47.0) | 55(45.0) | 1(1.0) | - |
| [130] | 20 | - | - | - | 20(100.0) | - | - | (72.0)† (40.0)‡ | - | - | - | - | - |
| [52] | 10 | 6(60.0) | - | 6(60.0) | - | 6(60.0) | 0(0.0) | 2(20.0) | 7(70.0) | 8(80.0) | 5(50.0) | 0(0.0) | - |
| [131] | 83 | 75(90.4) | 83(100.0) | - | 72(86.7) | 72(86.7) | - | - | 39(47.0) | 28(33.7) | 72(86.7) | 12(14.5) | 40(48.2) |
| [51] | 118 | 72(61.0) | - | - | 43(36.4) | 48(40.7) | 27(22.9) | 13(11.0) | 30(25.4) | - | 36(30.5) | 3(2.5) | 43(36.4) |
| [53] | 18 | 18(100.0) | 18(100.0) | 15(83.3) | 15(83.3) | 15(83.3) | 2(11.1) | 9(50.0) | 14(77.8) | 15(83.3) | 5(27.8) | 5(27.8) | - |
| [135] | 124 on admission | 80(64.5) | 124(100.0) | - | 79(63.7) | 79(63.7) | - | 17(13.7) | 39(31.5) | 49(39.5) | 72(58.1) | 0(0.0) | 39(31.5) |
| [135] | 195 during NICU stay | 184(94.4) | 195(100.0) | - | 184(94.4) | 184(94.4) | - | 18(9.2) | 90(46.2) | 99(50.8) | 169(86.7) | 0(0.0) | 95(48.7) |
| [54] | 77 | - | - | - | - | - | 21(27.3) | 76(98.7) | 65(84.4) | 68(88.3) | 77(100.0) | - | - |
| **Median (IQR)** |  | 63.0(59.5-80.9) | 100.0 | 58.6(52.5-77.5) | 63.7(40.4-86.7) | 61.9(42.1-85.9) | 17.0(8.3-24.0) | 12.4(6.7-35.0) | 42.9(29.8-73.9) | 50.9(45.6-80.8) | 50.0(36.8-86.7) | 4.9(1.4-25.0) | 42.9(36.4-48.2) |

* 100% for C3G ; ** for 3CG ; † Imipenem ; ‡ Ertapenem ; ¥ % for Non-ESBL producing *K. pneumoniae* strains

*AMX-C, Amoxicillin-clavulanic acid; AMX, amoxicillin; CRO, Ceftriaxone; CTX, Cefotaxime; CIP, Ciprofloxacin; CAZ, Ceftazidime; Cs, Colistin; Carb, Carbapanems; Fluorq, Fluoroquinolones ; SXT, Trimethoprim-sulfamethoxazole; GM, Gentamicin; AK, Amikacin; NA, Nalidixic acid*

**Table S3.** Resistance rates for *A. baumannii* isolates.

| **Study** | [111] | [117] | [59] | [121] | [57] | [56] | [52] | [66] | [132] | [60] | [53] | [55] | [58] | **Median (IQR)** |
| --- | --- | --- | --- | --- | --- | --- | --- | --- | --- | --- | --- | --- | --- | --- |
| ***Nb of isolates*** | **51** | **48** | **47** | **50** | **441** | **221** | **13** | **90** | **84** | **81** | **20** | **85 ABRI** | **64** |  |
| **Tetracycline** | - | - | - | - | 400(90.9) | 141(64.0) | - | - | - | - | - | - | 42(65.6) | 65.6(64.8-78.3) |
| **AMX-C** | - | - | - | - | - | - | - | - | - | - | 20(100.0) | - | - | 100.0 |
| **Ticarcillin** | 46(90.2) | - | 47(100.0) | - | 393(89.0) | - | 13(100.0) | 83(92.6) | 66(78.6) | 75(92.6) | 20(100.0) | - | 54(84.4) | 92.6(89.3-100.0) |
| **Piperacillin** | - | - | 47(100.0) | - | 386(87.6) | - | 13(100.0) | 83(92.6) | 68(81.0) | 75(92.6) | 20(100.0) | - | - | 92.6(90.1-100.0) |
| **Piperacillin/**  **tazobactam** | - | - | 47(100.0) | - | 349(79.2) | 212(96.0) | 13(100.0) | 83(92.6) | 22(26.2) | 70(86.4) | 20(100.0) | - | - | 94.3(84.6-100.0) |
| **Ticarcillin/**  **clavulanic acid** | - | - | 47(100.0) | - | 385(87.4) | - | - | - | 65(77.4) | 74(91.4) | 20(100.0) | - | 54(84.4) | 89.4(85.5-97.9) |
| **Ceftazidime** | 42(82.4) | - | 46(98.0) | - | 379(86.0) | 188(85.0) | 13(100.0) | 83(92.6) | 67(79.8) | 75(92.6) | 13(65.0) | - | 54(84.4) | 85.5(82.9-92.6) |
| **Ceftriaxone** | - | - | - | - | - | - | - | - | - | 72(88.9) | - | - | - | 88.9 |
| **Imipenem** | 35(68.6) | 34(71.0) | 27(57.4) | 37(74.0) | 336(76.2) | 168(76.0) | 13(100.0) | 85(94.4) | 9(10.7) | 23(28.4) | 18(90.0) | NR | 48(75.0) | 74.5(65.8-79.7) |
| **Amikacin** | 36(70.6) | - | 28(59.5) | - | 231(52.3) | 146(66.0) | (50.0) | 48(53.0) | 8(9.5) | 41(50.6) | 9(45.0) | 63(74.0) | 28(43.8) | 52.3(47.5-62.8) |
| **Gentamycin** | - | - | 47(100.0) | - | - | 172(78.0) | 11(83.3) | 85(94.4) | 67(79.8) | 60(74.1) | 18(90.0) | 80(94.0) | 56(87.0) | 87.0(79.8-94.0) |
| **Tobramycin** | - | - | 47(100.0) | - | 190(43.0) | 97(44.0) | 7(53.8) | 0(0.0) | 67(79.8) | 61(75.3) | 20(100.0) | 79(93) | 52(81.0) | 77.6(46.5-90.0) |
| **CIP** | - | - | 47(100.0) | - | 387(87.8) | 194(88.0) | 13(100.0) | 48(53.0) | 64(76.2) | 64(79.0) | 20(100.0) | 80(94.0) | 56(87.0) | 87.9(81.0-98.5) |
| **Levofloxacin** | - | - | - | - | - | - | - | - | - | - | 20(100.0) | - | 55(86.0) | 93.0(89.5-96.5) |
| **Rifampicin** | - | - | - | 43(86.0) | 142(32.1) | - | 6(46.1) | 48(53.0) | - | - | 19(95.0) | - | - | 53.0(46.1-86.0) |
| **Colistin** | - | - | 0(0.0) | 0(0.0) | 7(1,7) | 2(1.0) | 0(0.0) | 0(0.0) | - | - | 4(20.0) | 0(0.0) | - | 0.0(0.0-1.2) |
| **SXT** | - | - | 25(53.0) | - | 348(79.0) | 170(77.0) | 12(92.3) | 48(53.0) | 8(9.5) | - | 20(100.0) | 41(48.0) | 24(38.0) | 53.0(48.0-79.0) |
| **Cefepime** | - | - | 46(98.0) | - | 380(86.2) | - | - | - | - | 72(88.9) | - | - | 55(86.0) | 87.6(86.2-91.2) |
| **Netilmicin** | - | - | - | - | 146(33.1) | 31(14.0) | 6(46.1) | 0(0.0) | - | - | - | - | 46(71.0) | 33.1(14.0-46.1) |

*ABRI, Acinetobacter baumannii resistant to imipenem; NR, not recommended; AMX-C, Amoxicillin-clavulanic acid; CIP, Ciprofloxacin; SXT, Trimethoprim-sulfamethoxazole*

**Table S4.** Resistance rates for *S. pneumonia* isolates

| **Study** | [110] | [112] | [114] | [119] | [122] | [129] | [134] | **Median (IQR)** |
| --- | --- | --- | --- | --- | --- | --- | --- | --- |
| ***Nb of isolates*** | **150** | **313** † | **24** | **150** | **27** | **92** ‡ | **19** ¥ |  |
| **Penicillin G** | 52(34.7) | - | 2(8.3)* | 58(38.7) | 27(100.0) | 75(81.5) | 2(10.5) | 36.7(10.0-86.1) |
| **Amoxicillin** | 5(3.3) | 3(1.0) | 1(4.2) | 17(11.3) | 27(100.0) | - | 2(10.5) | 7.4(2.7-33.5) |
| **AMX-C** | - | - | - | - | 27(100.0) | - | 2(10.5) | - |
| **Cefotaxime** | 0(0.0) | - | - | 11(7.3) | - | - | - | - |
| **Ceftriaxone** | - | 3(1.0) | 0(0.0) | - | 10(37.0) | - | 2(10.5) | 5.8(0.3-30.4) |
| **Tetracycline** | - | 95(30.5) | - | 75(50) | - | 77(83.7) | - | 50.0(30.5-83.7) |
| **Erythromycin** | - | 45(14.4) | 4(16.6) | 93(62) | - | 92(100.0) | 4(21.0) | 21.0(15.5-81.0) |
| **Gentamycin** | - | - | - | 2(1.3) | 21(77.8) | - | - | - |
| **Ciprofloxacin** | - | - | - | 106(70.7) | 7(25.9)) | - | - | - |
| **Levofloxacin** | - | - | - | - | 5(18.5) | - | 2(10.5) | - |
| **Chloramphenicol** | - | 25(8.1) | 0(0.0) | 57(38) | - | - | - | 8.1(0.0-38.0) |
| **SXT** | - | 74(23.8) | 8(33.3) | 96(57.3) | - | 32(34.8) | 3(15.8) | 33.3(19.8-46.1) |

† Survey of 2006-2008; ‡ Erythromycin-resistant *S. pneumonia*; ¥ EUCAST susceptibility; * For MIC ≥ 2mg/L

*AMX-C, Amoxicillin-clavulanic acid; SXT, Trimethoprim-sulfamethoxazol*
