# Supplementary material for: Antimicrobial resistance among GLASS pathogens in Morocco: an epidemiological scoping review
Source: BMC Infect Dis. 2022 May 7;22:438. doi: 10.1186/s12879-022-07412-4 (PMC9077917; doi:10.1186/s12879-022-07412-4)
Supplement: Supplementary file 2 — Additional file 2: Appendix 1. GLASS specific antibiotic susceptibility testing against individual organisms (WHO, 2020). Appendix 2. Search strategy. Appendix 3. Joanna Briggs Institute’s critical appraisal checklist for studies reporting prevalence data. Appendix 4. Data extraction form [file 12879_2022_7412_MOESM2_ESM.docx]

# Appendices

## Appendix 1. GLASS specific antibiotic susceptibility testing against individual organisms (WHO, 2020)

| **Organism** | **Antibacterial class** | **Antibacterial agents that may be used for AST** |
| --- | --- | --- |
| *Escherichia coli* | Sulfonamides and trimethoprim | Co-trimoxazole |
|  | Fluoroquinolones | Ciprofloxacin or levofloxacin |
|  | Third-generation cephalosporins | Ceftriaxone, cefotaxime or Ceftazidime |
|  | Fourth-generation cephalosporins | Cefepime |
|  | Carbapenems | Imipenem, meropenem, ertapenem or doripenem |
|  | Polymyxins | Colistin |
|  | Penicillins | Ampicillin |
| *Klebsiella pneumoniae* | Sulfonamides and trimethoprim | Co-trimoxazole |
|  | Fluoroquinolones | Ciprofloxacin or levofloxacin |
|  | Third-generation cephalosporins | Ceftriaxone, cefotaxime or Ceftazidime |
|  | Fourth-generation cephalosporins | Cefepime |
|  | Carbapenems | Imipenem, meropenem, ertapenem or doripenem |
|  | Polymyxins | Colistin |
| *Acinetobacter* spp*.* | Tetracyclines | Tigecycline or minocycline |
|  | Aminoglycosides | Gentamicin and amikacin |
|  | Carbapenems | Imipenem, meropenem ordoripenem |
|  | Polymyxins | Colistin |
| *Staphylococcus aureus* | Penicillinase-stable beta-lactams | Cefoxitin |
|  | Penicillins | Oxacillin |
| *Streptococcus pneumoniae* | Penicillins | Oxacillin |
|  |  | Penicillin G |
|  | Sulfonamides and trimethoprim | Co-trimoxazole |
|  | Third-generation cephalosporins | Ceftriaxone |
|  |  | Cefotaxime |
| *Salmonella* spp. | Fluoroquinolones | Ciprofloxacin or levofloxacin |
|  | Third-generation cephalosporins | Ceftriaxone, cefotaxime or ceftazidime |
|  | Carbapenems | Imipenem, meropenem, ertapenem or doripenem |
| *Shigella* spp. | Fluoroquinolones | Ciprofloxacin or levofloxacin |
|  | Third-generation cephalosporins | Ceftriaxone, cefotaxime or Ceftazidime |
|  | Macrolides | Azithromycin |
| *Neisseria gonorrhoeae* | Third-generation cephalosporins | Cefixime or ceftriaxone |
|  | Macrolides | Azithromycin |
|  | Aminocyclitols | Spectinomycin |
|  | Fluoroquinolones | Ciprofloxacin |
|  | Aminoglycosides | Gentamicin |

## Appendix 2. Search strategy

**PubMed**

| **Item** | **Search terms** |
| --- | --- |
| #1 | "drug resistance, microbial"[MeSH Terms] OR "drug resistance, bacterial"[MeSH Terms] AND "Morocco"[MeSH Terms] |
| #2 | 1 AND Publication date from 2011/01/01 to 2021/12/20 ; Languages: Arabic, English, French, Spanish |

## Appendix 3. Joanna Briggs Institute’s critical appraisal checklist for studies reporting prevalence data

| **Items** | **Yes** | **No** | **Unclear** | **Not applicable** |
| --- | --- | --- | --- | --- |
| 1. Was the sample frame appropriate to address the target population? |  |  |  |  |
| 2. Were study participants sampled in an appropriate way? |  |  |  |  |
| 3. Was the sample size adequate? |  |  |  |  |
| 4. Were the study subjects and the setting described in detail? |  |  |  |  |
| 5. Was the data analysis conducted with sufficient coverage of the identified sample? |  |  |  |  |
| 6. Were valid methods used for the identification of the condition? |  |  |  |  |
| 7. Was the condition measured in a standard, reliable way for all participants? |  |  |  |  |
| 8. Was there appropriate statistical analysis? |  |  |  |  |
| 9. Was the response rate adequate, and if not, was the low response rate managed appropriately? |  |  |  |  |

## Appendix 4. Data extraction form

| **Article information** | - First author - Year of publication - Year(s) of data collection |
| --- | --- |
| **Study design and methods** | - Type of study - Study setting [hospital, primary healthcare, community] - Sample size - Age group [children <18 years; adults =>18 years] - Number of samples collected - Primary outcome measure |
| **Antimicrobial resistances** | - Pathogen(s) isolated - Laboratory methodology for pathogen identification - Laboratory methodology for determination of drug susceptibility - Criteria used for interpretation of resistances - AMR profile [antibiotic(s) tested; prevalence of AMR microorganisms] |
| **Additional comments** |  |
